# Exploring the Potential Observations Between Geomagnetic Activity and Cardiovascular Events: A Scoping Review

**DOI:** 10.7759/cureus.99851

**Published:** 2025-12-22

**Authors:** Jasen Belenko, Gabriel Cancel, Harvey N Mayrovitz

**Affiliations:** 1 College of Medicine, Nova Southeastern University Dr. Kiran C. Patel College of Osteopathic Medicine, Clearwater, USA; 2 Medical Education, Nova Southeastern University Dr. Kiran C. Patel College of Allopathic Medicine, Davie, USA

**Keywords:** cardiovascular disease, cosmic rays, geomagnetic activity, myocardial infarction, schumann resonance, solar activity, solar flares, space weather, stroke

## Abstract

Cardiovascular disease (CVD) is the leading cause of death globally, with a growing impact worldwide, yet the role of environmental exposures such as geomagnetic activity (GMA) is unclear. In recent years, environmental factors such as air pollution, extreme temperatures, and natural disasters have been recognized as triggers for cardiovascular events, prompting interest in other environmental influences. Geomagnetic activity (GMA), defined as fluctuations in Earth’s magnetic field driven by solar energy and charged particles, remains understudied due to challenges in its integration into epidemiologic research. This scoping review aimed to map the existing evidence on reported associations between geomagnetic activity and cardiovascular outcomes. A systematic literature search of PubMed, Web of Science, Excerpta Medica Database (EMBASE), and Cumulative Index to Nursing and Allied Health Literature (CINAHL) identified 1,718 articles published between 1964 and 2023. After removal of 147 duplicates and screening against predefined eligibility criteria, 36 studies were included in the final review. These studies examined adult populations, measured geomagnetic activity or related space-weather exposures (geomagnetic storms, solar proton events, high-speed solar wind, cosmic ray intensity, and Schumann resonances), and reported cardiovascular outcomes such as myocardial infarction, acute coronary syndrome, stroke, or mortality. The majority of studies (n = 28) reported significant correlations, while eight found no effect. The incidence of myocardial infarction and acute coronary syndrome increased during geomagnetic storms, solar proton events, and high-speed solar wind, with greater susceptibility observed in individuals with diabetes, metabolic syndrome, or prior cardiovascular disease. The risk of stroke increased with storm intensity, up to 52% during severe events, particularly among young adults. Low geomagnetic activity combined with high cosmic ray activity was consistently associated with increased myocardial infarction incidence and mortality, while more active solar conditions appeared protective. Overall, evidence suggests that geomagnetic and cosmic variability may coincide with cardiovascular risk; however, findings remain inconsistent, and many studies rely on ecological designs with uncontrolled factors that limit interpretation. Given that evidence is still emerging, these observations remain preliminary. Standardized prospective studies are necessary to determine underlying mechanisms and assess whether space weather monitoring could benefit cardiovascular risk prediction and public health preparedness.

## Introduction and background

Cardiovascular disease is the leading cause of death worldwide, responsible for approximately 18 million deaths each year [1]. Despite major strides in prevention and treatment, the global burden of cardiovascular disease continues to rise, with projections estimating a jump in prevalence from 598 million cases in 2025 to 1.14 billion by 2050, a trend partly driven by rising population-level risk factors that indicates the need to examine factors beyond those traditionally studied [2]. As traditional risk factors such as smoking, poor diet, and lack of physical activity have been extensively studied and targeted in public health initiatives, there is growing interest in understanding how environmental and external physical factors may also influence cardiovascular health [3]. Increasingly, exposures like air pollution, extreme temperatures, and natural disasters are recognized for triggering or worsening cardiovascular events through stress-response activation, systemic inflammation, oxidative stress, and immune activation [4]. These established environmental contributors support investigating additional, less-studied exposures that may also affect cardiovascular physiology.

One emerging area of interest is geomagnetic activity, a natural environmental phenomenon that remains largely absent from clinical and public health discussions. Geomagnetic activity refers to fluctuations in Earth’s magnetic field caused by solar energy and charged particles emitted by the Sun. Transported by the solar wind, these particles interact with Earth's magnetic environment and can lead to disturbances known as geomagnetic storms and substorms, which are tracked using standardized geomagnetic indices (e.g., Kp index, Ap index) [5-7]. Such disturbances are part of a larger system known as space weather, encompassing solar flares, coronal mass ejections, and other solar events that affect Earth’s upper atmosphere and magnetic field.

Although humans do not directly perceive geomagnetic activity, emerging research suggests it may influence cardiovascular health through subtle physiological mechanisms. These include disruptions in autonomic nervous system function, blood pressure regulation, and heart rate variability, processes known to affect cardiovascular risk [8]. Effects of a simulated Schumann resonance frequency have been reported to have a potential impact on skin blood flow [9]. Multiple studies have further linked periods of elevated geomagnetic activity to increased rates of myocardial infarction, stroke, and sudden cardiac death, though these associations are based largely on observational data [10].

To better understand these associations, several observational studies have investigated how geomagnetic disturbances may affect cardiovascular function in specific populations. In Athens, Greece, researchers found that heart rate decreased during periods of heightened geomagnetic activity in healthy individuals monitored with 24-hour electrocardiogram recordings [11]. Similarly, in Italy, outpatient data from a hypertension clinic showed that both systolic and diastolic blood pressure readings were significantly higher on days with stronger geomagnetic fluctuations, measured by the local k-sum index [12]. The potential relationship between geomagnetic variability and blood pressure regulation, including proposed autonomic and vascular mechanisms, has recently been reviewed [13]. At a broader population level, hospital data from Havana, Cuba, revealed a higher frequency of acute myocardial infarctions during periods of moderate to high geomagnetic activity, with a notable increase on the day after peak disturbance levels [14]. While study designs and settings vary, these consistent patterns across different regions suggest that geomagnetic activity may be a meaningful, yet underrecognized, factor in cardiovascular risk.

Despite growing interest, research on the relationship between geomagnetic activity and cardiovascular health remains limited and inconsistent. Studies differ in their methods, exposure definitions, outcome measures, and populations studied, making it challenging to draw clear or generalizable conclusions. Terminology is also used variably; terms like “solar storm,” “space weather,” and “geomagnetic disturbance” are often used interchangeably, despite having distinct meanings. These methodological and conceptual differences, along with the absence of a comprehensive synthesis, make it difficult to evaluate the overall strength of the evidence or identify gaps in knowledge.

To address this, the present scoping review aims to map the existing research on geomagnetic activity and cardiovascular events, summarize patterns and limitations in the literature, and highlight areas for future study.

## Review

Study design and search strategy

A scoping review was conducted to summarize current research on how geomagnetic activity may affect cardiovascular events and to identify areas where further studies are needed. A comprehensive literature search was conducted using four electronic databases: PubMed, Web of Science, Cumulative Index to Nursing and Allied Health Literature, and Excerpta Medica Database. Articles were identified during the search period from May 2025 to August 2025. The aim was to identify peer-reviewed primary studies that examine the relationship between geomagnetic or cosmic environmental activity and cardiovascular outcomes.

To cover a wide range of relevant research, a broad set of keywords was used. Cardiovascular-related terms included “stroke,” “ischemic stroke,” “hemorrhagic stroke,” “myocardial infarction,” “heart attack,” “acute coronary syndrome,” “cardiac arrest,” and “sudden cardiac death.” These were combined with geomagnetic and cosmic terms such as “geomagnetic storm,” “solar flare,” “solar activity,” “space weather,” “cosmic radiation,” “heliobiology,” and “Schumann resonance.” Boolean operators (“AND”/“OR”) were applied to combine these terms in various ways, increasing the search scope and capturing studies using different terminology for similar concepts. For example, a typical search combined (“stroke” OR “myocardial infarction” OR “cardiac arrest”) AND (“geomagnetic activity” OR “solar storm” OR “cosmic radiation”). This inclusive search strategy aimed to capture all potentially relevant studies for thorough screening and review.

Eligibility criteria

Inclusion criteria were established before the search to ensure consistent study selection. Studies were included if they were written in English, peer-reviewed, involved adult human participants, focused on cardiovascular outcomes (such as stroke, myocardial infarction, acute coronary syndrome, cardiac arrest, or cardiovascular mortality), and measured at least one type of geomagnetic or cosmic activity (e.g., geomagnetic storms or solar flares). Studies were excluded if they were review articles, abstracts without full-text access, non-human studies, did not primarily focus on geomagnetic or environmental exposures, or did not report cardiovascular outcomes.

Study selection process

**Figure 1:**
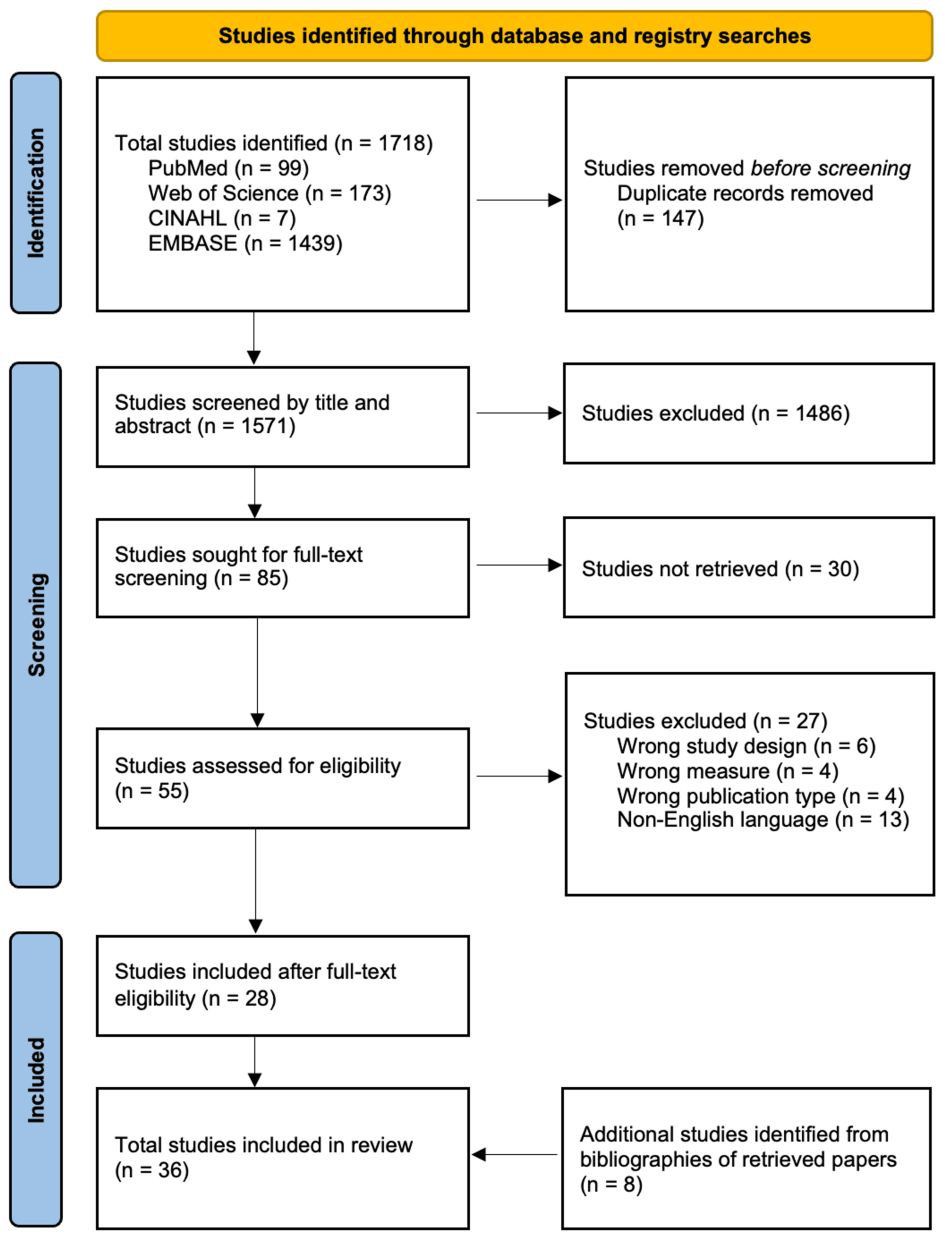
Search Approach The diagram is a Preferred Reporting Items for Systematic Reviews and Meta-Analyses (PRISMA) flowchart that details the study selection process. It illustrates how studies were identified, screened, excluded, and included, along with the reasons for exclusion at each stage.

The initial search retrieved 1,718 articles from the selected databases. After removing 147 duplicates, 1,571 articles remained. These were screened by title and abstract using the predefined eligibility criteria, resulting in the exclusion of 1,486 articles that lacked relevant cardiovascular or geomagnetic content. This left 85 articles for full-text review. Of these, 30 were excluded due to lack of full-text access. The remaining 55 articles were then evaluated in detail for relevance and compliance with the inclusion criteria. After full-text screening, 27 articles were excluded because of incorrect study design, outcome measures, or publication type. Ultimately, 28 studies met all inclusion criteria and were included in the final review. An additional eight studies were identified through the bibliographies of retrieved papers, resulting in a total of 36 studies included for review. Across all stages, the most common exclusion reasons were inappropriate study design and lack of relevance to geomagnetic or space-weather exposures. The complete selection process is illustrated in the Preferred Reporting Items for Systematic Reviews and Meta-Analyses (PRISMA) flowchart shown in Figure 1.

Data quality appraisal

Two investigators independently screened the full-text articles to determine study eligibility. Prior to screening, the authors established and agreed upon the inclusion criteria and data extraction guidelines to ensure consistency. Titles, abstracts, and full texts were systematically reviewed to identify relevant studies. Screening excluded articles with irrelevant populations or outcomes, no assessment of geomagnetic or space-weather exposures, or inappropriate study designs. The search identified 1,718 records, and after duplicate removal and all screening stages, 28 studies met the inclusion criteria; an additional eight studies were identified through reference screening, yielding a total of 36 included studies. Any discrepancies in study selection or data extraction were resolved through discussion until consensus was reached.

From each study, the following data were extracted: reference, country, study aim, sample size, study design, and main findings. This information was summarized in Table 1 to provide a clear overview of the included studies and their key characteristics. The methodological quality and relevance of the included studies were then assessed using the Joanna Briggs Institute (JBI) Critical Appraisal Tools, which evaluate the reliability and potential bias of various study designs, including case-control, cohort, and randomized controlled trials [15]. No studies were excluded based on quality assessment, and all 36 studies were included in the final analysis.

**Table 1:** Summary of Included Articles Column 1 contains the first author, year of publication, and reference number. Column 2 contains the country of the study population. Column 3 contains the study aim. Column 4 contains the number of subjects in the study. Column 5 contains the study design. Column 6 contains the main findings of the study. The following abbreviations are used in this table: The K index refers to a measure of geomagnetic activity; GMF: geomagnetic field; CVD: cardiovascular disease; AMI: acute myocardial infarction; STEMI: ST-elevation myocardial infarction; SPEs: solar proton events; LMF: local magnetic fields; GMS: geomagnetic storms; ICMEs: interplanetary coronal mass ejections; MI: myocardial infarction; HSSW: high-speed solar wind; ACS: acute coronary syndrome; DM: diabetes mellitus; MS: metabolic syndrome; NO₂: nitrogen dioxide; GMD: geomagnetic disturbances; and QBO: quasi-biennial oscillation.

Reference	Country	Study Aim	Sample Size	Study Design	Main Findings
Vencloviene (2011) [16]	Lithuania	To investigate whether geomagnetic activity modifies the association between short-term NO2 exposure and emergency hospitalization for ACS	6,594	Case-crossover study	NO2 exposure increased ACS risk in younger patients, and combined exposure with high geomagnetic activity further amplified this risk, suggesting geomagnetic activity intensifies the effect of traffic-related air pollution on ACS
Vencloviene (2013) [17]	Lithuania	To assess associations between geomagnetic activity, solar proton events, solar flares, meteorological factors, and patient characteristics in STEMI admissions	1,979	Retrospective observational study	Geomagnetic storms and solar proton events significantly increase STEMI risk, especially in patients with stable angina and renal disease
Vencloviene (2016) [18]	Lithuania	To evaluate how geomagnetic storms, solar proton events, and X-class solar flares affect emergency heart attack admissions during solar activity phases	12,330	Retrospective time-series study	MI admissions increased significantly on days with high proton flux and during geomagnetic storms combined with SPEs, with elevated risk also observed before and after these events
Vencloviene (2017) [19]	Lithuania	To assess the impact of GMS, SPEs, and HSSW on the risk of ACS in patients with DM and MS	1,548	Retrospective observational cohort study	GMS, SPEs, and HSSW increase ACS risk, especially in patients with DM and MS during and shortly after these events
Vaičiulis (2021) [20]	Lithuania	To examine how geomagnetic storms and other space weather events relate to acute myocardial infarction cases and ischemic heart disease deaths	15,072	Retrospective time-series observational study	Risk of AMI and IHD mortality increased from days before to after geomagnetic storms, especially in spring to autumn; high-speed solar wind had the strongest impact on the event day, while solar flares during storms and ICMEs were linked to higher AMI and IHD deaths several days later
Kleimenova et al. (2007) [21]	Russia, Bulgaria	To investigate the effects of geomagnetic pulsations (Pc1) on myocardial infarctions and their seasonal variation	85,800	Ecological Time-Series Study	Myocardial infarctions and sudden deaths showed a seasonal pattern, peaking in winter and declining in summer. Pc1 geomagnetic pulsations followed a similar seasonal trend and were associated with increases in MI, while magnetic storms showed stronger associations in winter than in summer
Vencloviene (2013) [22]	Lithuania	To investigate the impact of solar proton events and geomagnetic storms on emergency hospitalizations for first-time myocardial infarction with and without ST segment elevation	2,008	Case-crossover study	Solar proton events combined with geomagnetic storms increase the risk of MI with ST elevation, while MI without ST elevation risk rises after solar proton events followed by geomagnetic storms; geomagnetic storms alone do not increase MI risk without ST elevation
Jarusevicius (2018) [23]	Lithuania	To assess the relationship between changing geomagnetic field strength and weekly STEMI incidence	703	Retrospective observational study	Weekly STEMI incidence correlated with local geomagnetic field strength across frequencies, with associations varying by age and sex, indicating different individual sensitivities
Qammar (2023) [24]	Lithuania	To investigate the long-term relationship between local magnetic fields (Schumann resonances) and AMI	Not specified	Retrospective ecological time-series study	AMI incidence showed a long-term association with LMF variations, especially in the beta and gamma Schumann resonance frequencies
Vencloviene (2020) [25]	Lithuania	To investigate associations between daily AMI rates and weather, GA, solar wind variables, and QBO phases	Not specified	Population-based longitudinal observational study	High geomagnetic activity, stronger solar wind, and QBO phases influence daily AMI risk, with geomagnetic storm effects increasing during the east QBO phase and decreasing during the west phase
Stoupel (2007) [26]	Azerbaijan	To analyze daily geomagnetic, cosmic ray, and solar activity associated with acute myocardial infarction and pre-admission mortality	5,359	Retrospective ecological time-series study	AMI incidence increased during periods of low geomagnetic activity and elevated cosmic ray activity, with pre-admission mortality peaking under these same conditions
Stoupel (1997) [27]	Israel, Lithuania	To study how proton flux, solar activity, and geomagnetic activity affect the number of total deaths and heart-related deaths each month	23,600	Retrospective ecological time-series study	Proton flux increased as solar and geomagnetic activity decreased, showing a significant association with changes in total and cardiovascular mortality
Stoupel (2004) [28]	Lithuania	To investigate the monthly relationships between solar, geomagnetic, and cosmic ray activity and mortality rates, focusing on differences between genders	504,243	Retrospective ecological time-series study	Mortality rates were influenced by solar, geomagnetic, and cosmic ray activity, with notable differences between genders
Stoupel (2007) [29]	Lithuania	To examine long-term monthly relationships between death counts and cosmophysical activity by cause and gender	630,205	Retrospective ecological time-series study	Total deaths increased with cosmic ray activity and decreased with solar activity, especially in men, while stroke deaths grew in cardiovascular mortality and geomagnetic activity showed minor links to specific causes
Bellossi (1985) [30]	France	To examine the relationship between geomagnetic activity and myocardial infarction	747	Retrospective observational cohort study	No significant correlation between geomagnetic activity (K index) and myocardial infarction incidence
Morimoto et al. (2020) [31]	Japan	To evaluate the short-term effects of geomagnetic storms on human health by detecting both positive and negative responses	Not specified	Ecological time-series study	Geomagnetic storms were associated with decreases in myocardial infarction and acute heart failure, but with increases in cerebral infarctions
Messner (2002) [32]	Sweden	To examine the link between geomagnetic activity (aurora borealis) and AMI incidence	510,000	Retrospective observational study	No significant link was found between geomagnetic activity and fatal or non-fatal AMI, sudden deaths, or chest pain without myocardial damage
Podolská et al. (2018) [33]	Czech Republic	To assess the influence of geomagnetic and ionospheric factors on cardiovascular mortality and compare the predictive value of solar and ionospheric parameters.	6,573	Ecological time-series study	No direct association was found between geomagnetic indices and AMI or stroke mortality. In contrast, ionospheric factors better explained mortality patterns, suggesting cardiovascular deaths may be influenced indirectly through ionospheric activity
Fogel (1964) [34]	USA	To examine acute myocardial infarction (AMI) incidence and its relation to weather and solar activity	1,663	Retrospective observational study	No significant links were found between AMI and weather or sunspots, with AMI cases peaking in spring and women experiencing higher fatality rates, while sunspot activity showed a weak, non-significant correlation with non-AMI deaths
Shaposhnikov (2014) [35]	Russia	To assess the effects of air temperature, barometric pressure, and geomagnetic activity on hospital admissions for myocardial infarction and stroke	3,929	Retrospective time-series study	Geomagnetic storms, low or falling pressure, and temperature extremes were linked to increased hospitalizations for myocardial infarction and stroke, with strokes more sensitive to weather changes
Ozheredov et al. (2010) [36]	Russia	To assess the relative contributions of geomagnetic activity and weather factors to acute myocardial infarction and stroke morbidity	3,929	Ecological observational study	Geomagnetic activity was linked to AMI and stroke, but weather factors were stronger predictors
Shumilov et al. (2016) [37]	Russia	To compare the influence of geomagnetic disturbances with socioeconomic and environmental factors on long-term cardiovascular mortality trends in high-latitude regions	9,057	Retrospective ecological time-series study	Cardiovascular mortality showed seasonal peaks that coincided in part with geomagnetic activity, but was driven mainly by socioeconomic factors, with geomagnetic influences playing a secondary role
Feigin (2014) [38]	New Zealand, Australia, UK, France, Sweden	To investigate the impact of geomagnetic storms on stroke risk	11,453	Time-stratified case-crossover study	Geomagnetic storms were linked to higher stroke risk, especially in people under 65, with risk increasing as storm intensity grew
Vencloviene (2021) [39]	Lithuania	To investigate the relationship between space weather events and daily occurrences of ischemic and hemorrhagic strokes	8,226	Time-stratified case-crossover study	Intense geomagnetic storms and solar events are associated with elevated risks of hemorrhagic and ischemic strokes, while periods of low geomagnetic activity also correspond to increased stroke risk
Feigin (2000) [40]	Russia	To investigate the association between weather conditions and the occurrence of stroke and its subtypes	2,208	Population-based observational	Ischemic stroke was linked to low temperature and air pressure, intracerebral hemorrhage to mild temperature, and no weather associations were found for subarachnoid hemorrhage
Vieira (2019) [41]	USA	To investigate the association between short-term GMD and daily mortality from all causes, CVD, MI, and stroke	44,220,261	Multi-city ecological time-series study	GMD was linked to higher total, CVD, and MI deaths, especially in fall and winter, with a stronger effect than common air pollution and no clear link to stroke deaths
Chai (2023) [42]	Global (204 territories)	To examine correlations between geomagnetic field (GMF) characteristics and global cardiovascular disease (CVD) occurrence	Not specified	Longitudinal ecological study	Both stable and unstable GMF were associated with CVD, with a significant positive correlation between stable GMF intensity and CVD incidence, even after adjusting for income and time trends
Stoilova (2008) [43]	Bulgaria	To investigate the impact of geomagnetic activity on cardiovascular function and heart attack outcomes in healthy individuals and patients	1,219	Prospective repeated-measures study / Retrospective cohort study	Geomagnetic disturbances were associated with changes in diastolic and pulse pressure, showing limited early significance; hospital data indicated possible links to heart attack admissions and deaths
Amiranashvili et al. (2002) [44]	Georgia	To examine the relationship between space weather (geomagnetic activity and cosmic rays) and cardiovascular mortality	Not specified	Retrospective ecological time-series study	Cardiovascular deaths increased during low solar activity, with higher mortality in older adults and during the cold season
Dimitrova et al. (2009) [45]	Bulgaria	To investigate the relationship between solar and geomagnetic activity (GMA) and acute myocardial infarction (AMI) morbidity and mortality	Not specified	Ecological time-series study	AMI morbidity and mortality increased before, during, and after geomagnetic storms, with stronger effects from storms caused by magnetic clouds than from high-speed solar wind or quiet periods
Styro (2015) [46]	Lithuania	To investigate the relationship between cosmic ray flux, sunspot number, air temperature, and CVD incidence	250,000	Retrospective ecological time-series study	Decreases in cosmic ray flux were strongly linked to increases in cardiovascular disease shortly after, with high prediction accuracy; no significant connections were found with sunspot number or air temperature
Shumilov (2020) [47]	Russia	To assess the impact of space-weather factors, especially geomagnetic activity and solar proton events (SPEs), on suicide and CVD mortality	9,965	Retrospective ecological time-series study	Cardiovascular disease mortality exhibited periodic patterns corresponding to solar cycles; however, socioeconomic factors exerted a significantly greater influence on mortality rates than geomagnetic activity
Vencloviene (2014) [48]	Lithuania	To examine the link between GMS, SPEs, and solar flares around hospital admission and 2-year survival in ACS patients	1,413	Observational cohort study	Elevated geomagnetic activity shortly after admission increased cardiovascular death risk, with stronger effects in women and older patients, especially when combined with SPEs or solar flares
Samsonov et al. (2014) [49]	Russia	To investigate how geomagnetic activity and substorms influence seasonal patterns of myocardial infarction incidence in subauroral latitudes	711	Observational ecological time-series study	Myocardial infarction incidence increased during geomagnetic maxima, coinciding with night-time substorms and geomagnetic pulsations
Dimitrova et al. (2009) [50]	Bulgaria, Azerbaijan	To investigate the relationship between geomagnetic storms and daily acute myocardial infarctions (AMI)	5,671	Ecological time-series study	Acute myocardial infarction incidence was significantly higher on geomagnetic storm days, with stronger effects in women and in adults over 70
Mendoza & Diaz-Sandoval (2004) [51]	Mexico	To evaluate the impact of solar activity and geomagnetic disturbances on myocardial infarction deaths and to assess biological rhythms in their occurrence	129,917	Ecological time-series study	Myocardial infarction deaths increased during geomagnetic storms, with the strongest effects in elderly males and during solar maximum. A 7-day biological rhythm in MI deaths was observed during high solar activity but not during solar minimum
Stoupel & Shimshoni (1991) [52]	Israel	To examine whether hospital mortality varies with monthly solar and geomagnetic activity	15,601	Retrospective ecological time-series study	Hospital deaths overall increased with solar activity, while cardiovascular deaths (excluding MI) were negatively associated with geomagnetic activity

Results

The review included 36 studies published between 1964 and 2023, comprising ecological, time-series, case-crossover, and cohort designs. Out of the 36 articles, 28 found a positive or inverse correlation between geomagnetic activity and cardiovascular events, while eight concluded that geomagnetic activity did not influence cardiovascular outcomes.

Geomagnetic activity and myocardial infarction

Several studies have investigated the relationship between geomagnetic activity and myocardial infarction, although findings have been inconsistent across different regions and study designs. Multiple investigations reported an increased cardiovascular risk during periods of geomagnetic disturbance. High geomagnetic activity intensified the cardiovascular effects of nitrogen dioxide exposure in younger adults and showed a synergistic influence on myocardial infarction risk [16]. Emergency admissions for myocardial infarction increased during geomagnetic storms and solar proton events, with particularly strong effects among individuals with pre-existing conditions such as stable angina or chronic kidney disease [17]. Subsequent analyses confirmed elevated risk of myocardial infarction and acute coronary syndrome during and shortly after solar proton events, magnetic storms, and high-speed solar wind [18,19]. Increases in myocardial infarction-related mortality were also observed in the days surrounding geomagnetic disturbances, with the strongest seasonal peaks in spring and autumn when vascular and autonomic responses tend to be more variable as the body adapts to shifting environmental conditions [20]. However, other publications suggest that seasonal peaks of myocardial infarction occur during winter due to higher associations with magnetic storms and lower associations during summer [21]. Solar proton events combined with geomagnetic storms increased hospital admissions for both ST-elevation myocardial infarction and non-ST-elevation myocardial infarction, with risk differing by subtype and showing greater effects at higher latitudes, where stronger geomagnetic disturbances expose populations to greater magnetic field fluctuations [22].

Frequency-specific associations have also been reported. Schumann resonances, which are natural electromagnetic waves within the Earth-ionosphere cavity, have been shown to exhibit distinct associations with cardiovascular outcomes [23]. Weekly incidence of ST-elevation myocardial infarction increased with fluctuations in the higher-frequency gamma and beta bands, whereas lower-frequency bands demonstrated weaker or inverse associations [23]. Long-term variation in myocardial infarction incidence correlated with beta and gamma Schumann resonance frequencies, while prolonged fluctuations in geomagnetic intensity were more consistently associated with myocardial infarction than acute storm activity, suggesting a cumulative effect of chronic exposure [24]. Acute myocardial infarction risk was associated with geomagnetic storms, solar wind speed and pressure, and the quasi-biennial oscillation phase (a roughly two-year cycle of equatorial stratospheric wind shifts), with stronger effects in winter and during stream interaction regions [25].

Cosmic ray activity has been identified as an additional factor influencing cardiovascular outcomes. Periods of low geomagnetic activity combined with elevated cosmic ray activity were associated with higher myocardial infarction incidence and pre-hospital mortality [26]. Cosmic ray exposure has been linked to oxidative stress, increased blood viscosity, and autonomic imbalance, mechanisms that may contribute to cardiovascular instability [27,28]. Total mortality, particularly among men, was positively correlated with higher cosmic ray counts, supporting cosmic ray activity as a potential modifier of cardiovascular risk [29]. Reduced solar conditions weaken magnetic shielding and allow more cosmic rays to reach Earth. Together, these findings suggest that both geomagnetic disturbances and the increased cosmic particle flux during these periods may influence cardiovascular health.

Not all investigations demonstrated consistent associations. An early study reported no increase in geomagnetic fluctuations on myocardial infarction days [30]. In Japan, geomagnetic storms were linked to a decreased incidence of myocardial infarction and acute heart failure [31]. In Sweden, however, an initial association between geomagnetic activity and cardiovascular outcomes was not confirmed in follow-up analyses using the same methodology [32]. Research from Central Europe found no direct correlation between geomagnetic activity and acute myocardial infarction or stroke mortality but suggested an indirect link through interactions between space weather and ionospheric charge concentrations [33]. Other investigations of sunspot cycles and meteorological conditions similarly found no direct link with myocardial infarction, although seasonal peaks, particularly in spring, were noted [34]. A study from Moscow reported modest associations between geomagnetic storms and myocardial infarction hospitalizations, but barometric pressure and temperature emerged as stronger predictors [35,36]. Socioeconomic stresses have also been identified as stronger determinants of myocardial infarction deaths, while geomagnetic effects may contribute but are not dominant [37]. These discrepancies highlight the modifying role of geography, population characteristics, and environmental exposures, which complicate efforts to isolate the independent impact of space weather on cardiovascular outcomes.

Geomagnetic activity and other cardiovascular outcomes

Although myocardial infarction has been the most frequently studied outcome, several investigations have reported associations between geomagnetic activity and other cardiovascular diseases. Geomagnetic storms were associated with a 19% overall increase in stroke incidence, with the strongest effects observed in individuals younger than 65 years. The risk of stroke rose progressively with storm intensity, reaching a 52% higher risk during strong or severe/extreme storms [38]. In Lithuania, strong or severe geomagnetic activity was linked to higher rates of subarachnoid hemorrhage (58%) and hemorrhagic stroke (30%), while low geomagnetic activity correlated with a greater incidence of intracerebral hemorrhage, hemorrhagic stroke, and ischemic stroke [39]. In Siberia, low temperature and air pressure predicted ischemic stroke, mild temperature predicted intracerebral hemorrhage, and no associations were found for subarachnoid hemorrhage [40]. By contrast, no significant relationship between stroke and geomagnetic disturbances was identified in Brazil, although clear seasonal patterns were observed for myocardial infarction deaths, total deaths, and cardiovascular disease, particularly during spring and fall [41].

Regional variation in geomagnetic field parameters has also been examined. In China, total geomagnetic field intensity was positively correlated with cardiovascular mortality, whereas horizontal geomagnetic field intensity showed an inverse association, possibly because stable horizontal components create less short-term geomagnetic variation and reduce physiologic stress [42]. This shift from positive to negative associations was also observed across latitudes, with results differing between polar regions and the equator [42]. In Bulgaria, elevated geomagnetic field intensity was associated with higher blood pressure, more frequent cardiovascular complaints, and increased myocardial infarction incidence and mortality through subclinical physiologic stress pathways involving early autonomic and vascular responses [43-44]. A separate Bulgarian study also found that stronger geomagnetic field intensity was linked to additional blood pressure changes, more subjective complaints, and higher myocardial infarction rates, reinforcing the adverse cardiovascular impact of geomagnetic disturbances [45]. Decreases in hard cosmic ray flux were noted to precede abrupt rises in cardiovascular events [46]. In Russia, variations in solar proton events and geomagnetic disturbances were associated with cardiovascular mortality, though the observed effects were weaker than those of socioeconomic determinants, underscoring the modifying role of broader contextual factors [47].

Influence of specific geomagnetic and space weather factors

Cardiovascular outcomes have been shown to vary in relation to geomagnetic activity and other space weather phenomena, including geomagnetic storms, solar proton events, high-speed solar wind, solar flares, and proton flux. The most pronounced effects were observed when multiple disturbances occurred concurrently. Geomagnetic storms in combination with solar proton events were associated with a marked increase in the incidence of ST-elevation myocardial infarction, whereas solar proton events alone were followed by a higher incidence of non-ST-elevation myocardial infarction with a two-day lag [48]. Cardiovascular mortality also increased when geomagnetic activity coincided with solar proton events and solar flares, with post-event survival disproportionately reduced among women and older adults [18]. Active or stormy geomagnetic conditions within days of hospital admission increased the hazard of cardiovascular death in patients with acute coronary syndromes, with stronger effects in women and adults over 70 years old, and mortality risk was further amplified when geomagnetic storms overlapped with solar proton events or solar flares [22]. Night magnetospheric substorms were linked with increases in geomagnetic activity, which in turn coincided with higher incidences of myocardial infarction [49].

Hospital admissions for myocardial infarction were significantly higher on days of elevated proton flux, and this risk was further amplified when high flux overlapped with geomagnetic storms and solar proton events, with variation observed across solar cycle phases [18]. Within the same cohort, geomagnetic storms, solar proton events, and high-speed solar wind streams were each associated with an increased incidence of acute coronary syndrome among patients with diabetes mellitus and metabolic syndrome, suggesting that cardiometabolic comorbidities confer vulnerability [19]. Combined solar proton events and geomagnetic storms were also linked to a higher incidence of first-time ST-elevation myocardial infarction among patients with cardiovascular, renal, or pulmonary disease, whereas solar proton events alone heightened risk in those with stable angina [17]. The incidence of acute myocardial infarction was elevated during periods of strong geomagnetic activity, high-speed solar wind intensity, and the east phase of the quasi-biennial oscillation, with geomagnetic effects being particularly pronounced under these atmospheric conditions [25]. The concurrence of geomagnetic storms with high-speed solar wind streams produced the largest increases in both acute myocardial infarction and ischemic heart disease risk [20]. Conversely, low geomagnetic activity, combined with elevated cosmic ray activity, was consistently associated with higher acute myocardial infarction incidence and cardiovascular mortality. In contrast, periods of stronger geomagnetic activity and higher solar activity corresponded with a reduction in cosmic ray activity-related risk, consistent with the protective role of solar magnetic shielding against cosmic particle exposure [26-29].

Effect modifiers and population subgroups

Differences in cardiovascular response to geomagnetic activity have been documented across demographic and clinical subgroups, with sex emerging as a notable modifier. During geomagnetic storms, acute myocardial infarction incidence was significantly increased in women and the elderly [50]. Women demonstrated higher rates of ST-elevation myocardial infarction during periods of high-frequency geomagnetic fluctuations, whereas men exhibited weaker or inverse associations across the same spectral bands [23]. These sex-specific patterns may reflect hormonal and autonomic influences that alter vascular responsiveness and modulate cardiovascular reactions to external stressors. In contrast to previous research, increasing geomagnetic disturbances were associated with myocardial infarction deaths, with the strongest effects observed in elderly males during solar maximum [51]. Mortality studies further indicated that geomagnetic activity was strongly associated with cardiovascular and suicide-related deaths in men, suggesting sex-specific variability in vulnerability to magnetic field disturbances [27,29].

Clinical comorbidities have also been shown to influence cardiovascular outcomes during periods of space weather activity. Patients with diabetes experienced nearly a twofold increase in acute coronary syndrome during episodes of high-speed solar wind streams, particularly when these events coincided with geomagnetic storms or solar proton events [19]. Comparable effects were observed in individuals with metabolic syndrome, where pre-existing vascular dysfunction and systemic inflammation may increase sensitivity to external stressors. Age further influenced susceptibility: adults younger than 65 demonstrated greater myocardial infarction risk when traffic-related air pollution occurred alongside geomagnetic disturbances [16], while adults older than 70 exhibited higher cardiovascular mortality during geomagnetic disturbances, though outcomes in this group were also shaped by social determinants such as healthcare access and income level [47].

Seasonal variation introduced an additional dimension to the relationship between geomagnetic activity and cardiovascular outcomes. Mortality from heart disease consistently rose during the fall and winter months and remained significantly associated with geomagnetic disturbances even after adjustment for air pollution [38]. Seasonality emerges as a consistent modifier, with colder months heightening physiologic stress through increased blood pressure and sympathetic activation. Notably, this effect was not observed for stroke mortality, suggesting that coronary circulation may be more sensitive to magnetic field fluctuations than cerebrovascular pathways.

Mortality and hospital admissions related to geomagnetic activity

Fluctuations in geomagnetic activity, cosmic ray activity, and solar activity have been linked to variation in cardiovascular and overall mortality, with outcomes differing according to the combination of exposures. Myocardial infarction and overall hospital death were positively correlated with geomagnetic activity [52]. However, non-myocardial infarction cardiovascular hospital death was negatively correlated with geomagnetic activity [52]. Periods of low solar activity and geomagnetic activity accompanied by elevated proton flux were associated with increased cardiovascular mortality, consistent with greater penetration of high-energy cosmic particles into the atmosphere [30]. Broader health effects were subsequently reported, with space weather variability associated not only with cardiovascular outcomes but also with cancer, suicide, and accidents; among these, ischemic heart disease mortality demonstrated the strongest and most consistent relationship with geomagnetic activity [26]. Elevated cosmic ray activity in the setting of suppressed geomagnetic activity was followed by a higher incidence of acute myocardial infarction and increased pre-admission mortality, indicating that cosmic ray activity may accelerate ischemic onset and increase the likelihood of fatal outcomes before hospital care [24]. Mortality trends revealed opposing influences, with cosmic ray activity generally increasing overall risk and geomagnetic activity showing a more selective association, particularly with cerebrovascular outcomes such as stroke and with external causes including traffic accidents [29].

Hospital admissions for myocardial infarction and stroke also increased during geomagnetic storms, with stroke incidence showing heightened sensitivity under concurrent meteorological stressors such as low barometric pressure and abrupt temperature changes, both of which can destabilize hemodynamics and vascular tone [35]. Cardiovascular mortality demonstrated cyclical variation in alignment with the 11-year solar cycle and its second harmonic, with risk influenced by both proton flux intensity and the frequency of geomagnetic storms [47]. In Portugal, mortality from all causes, as well as from cardiovascular disease and myocardial infarction specifically, rose on geomagnetically disturbed days even after adjustment for seasonal variation, meteorological parameters, and fine particulate matter; these associations persisted across sex and age strata [38]. Additional observations showed that decreases in cosmic ray flux frequently preceded surges in cardiovascular events, suggesting that abrupt shifts in particle exposure may act as triggers for acute outcomes [46]. Variability in Schumann resonance frequencies, electromagnetic oscillations within the Earth-ionosphere cavity, was also correlated with acute myocardial infarction, further implicating atmospheric electromagnetic fluctuations as contributors to cardiovascular stress [24].

Discussion

Cardiovascular outcomes showed notable variation during periods of geomagnetic disturbance, with the most pronounced effects occurring when multiple space weather phenomena coincided. The overlap of geomagnetic storms with solar proton events or high-speed solar wind produced significant increases in myocardial infarction, acute coronary syndrome, and cardiovascular mortality [17-20]. These overlapping disturbances likely generated greater biological stress than single events by intensifying both electromagnetic fluctuations and particle exposure. Seasonal peaks in myocardial infarction-related mortality during spring and autumn [20] further indicate that periods of unstable geomagnetic activity amplify cardiovascular risk, potentially through interactions with other seasonal stressors such as abrupt temperature changes or infectious disease burden. Low geomagnetic activity, combined with elevated cosmic ray activity, was consistently linked to higher myocardial infarction incidence and pre-hospital mortality. In contrast, stronger solar activity appeared to mitigate these effects, consistent with the shielding role of solar magnetic fields in reducing cosmic particle penetration into the Earth’s atmosphere [26-29].

Beyond storm intensity, the frequency and duration of geomagnetic fluctuations were found to be important contributors to cardiovascular risk. Sustained disturbances were more consistently associated with myocardial infarction than short-lived events, suggesting that chronic exposure exerts a cumulative strain on physiological systems. These effects likely arise from mechanisms such as altered autonomic regulation, endothelial dysfunction, impaired vascular tone, and persistent low-grade inflammation [23,24,27,28]. The influence of geomagnetic variability extended to cerebrovascular outcomes. Geomagnetic storms were associated with a 19% overall increase in stroke incidence, with risk rising proportionally with storm intensity and most evident among individuals younger than 65 years [38]. In Lithuania, strong or severe geomagnetic activity was associated with higher rates of subarachnoid hemorrhage and hemorrhagic stroke, while low geomagnetic activity was linked to an increased incidence of both ischemic and hemorrhagic events [39]. In Siberia, low temperature and air pressure predicted ischemic stroke, while mild temperature predicted intracerebral hemorrhage, with no associations found for subarachnoid hemorrhage [40]. However, studies from Brazil did not identify a significant relationship between geomagnetic disturbances and stroke, although seasonal peaks in myocardial infarction and overall cardiovascular mortality persisted [41]. The finding that stroke incidence rose most sharply when geomagnetic disturbances coincided with abrupt meteorological shifts such as low barometric pressure or rapid temperature changes [35] suggests that geomagnetic variability may act as an additive burden in individuals already physiologically stressed.

Population characteristics and clinical conditions further modified susceptibility. Women exhibited higher rates of ST-elevation myocardial infarction during periods of high-frequency fluctuations, while men showed stronger associations between geomagnetic activity and cardiovascular or suicide-related mortality [23,27,29]. Patients with diabetes or metabolic syndrome faced nearly double the risk of acute coronary syndrome during episodes of high-speed solar wind and geomagnetic storms [19], likely reflecting underlying vascular dysfunction and systemic inflammation. Older adults, particularly those over 70, also experienced elevated cardiovascular mortality during geomagnetic disturbances, although socioeconomic determinants such as healthcare access played a role in shaping outcomes [47]. Seasonal analyses further reinforced these findings, showing that cardiovascular mortality was consistently elevated during fall and winter and remained significantly associated with geomagnetic activity even after adjusting for air pollution [41].

Not all investigations reported consistent effects. Early studies found no significant changes in geomagnetic activity on myocardial infarction days [30], and subsequent analyses from Sweden failed to replicate the initially positive associations when re-examined using the same methodology [32]. In Moscow, barometric pressure and temperature emerged as stronger predictors of cardiovascular risk than geomagnetic indices [35]. These discrepancies likely reflect regional variation in geomagnetic field strength, differences in population susceptibility, and the confounding influence of environmental exposures. Methodological variability further contributed to these discrepancies, as studies used different geomagnetic indices (e.g., Kp, Ap, Dst, local magnetometer readings), making it difficult to separate true geographic effects from differences in measurement. They also highlight the limitations of the predominantly ecological and observational designs in this field, which restrict causal inference and leave mechanistic pathways incompletely understood.

Collectively, the evidence suggests that both geomagnetic and cosmic activity have a quantifiable impact on cardiovascular events and that myocardial infarction and stroke are most consistently influenced. The coincidence of two or more space weather events, spectral frequency, and chronicity of geomagnetic variation, and the presence of vulnerable subgroups all appear to be significant in determining risk. While results have been inconsistent across geographical sites, the trend suggests that space weather may be an environmental risk factor for cardiovascular disease. Multicenter, prospective studies correlating standardized cardiovascular endpoints with high-resolution tracking of geomagnetic and atmospheric disturbances would be a high-priority follow-up activity. Mechanistic studies are also necessary to describe how electromagnetic and cosmic variation affects autonomic regulation, vascular tone, and inflammatory processes. Lastly, incorporating space weather into cardiovascular surveillance systems can provide early warning of excess cardiovascular events and strengthen public health preparedness during periods of high geomagnetic activity.

## Conclusions

Though the extent and nature of this relationship are uncertain, current evidence suggests that variations in the Earth's magnetic and cosmic environment may coincide with changes in cardiovascular disease rather than indicate a causal link. Observations involving myocardial infarction and stroke are most frequently reported; however, distinguishing geomagnetic influences from environmental and socioeconomic factors remains difficult, as shown by variation across studies. These inconsistencies indicate that causality cannot be inferred, and any potential effects of space weather likely operate through multiple mechanisms moderated by sex, age, season, location, weather, and socioeconomic factors.

Future studies should transition from ecological to prospective and mechanistic designs to determine whether space weather is a genuine cardiovascular risk factor. Verifying such a link could enhance cardiovascular prevention and preparedness, as geomagnetic monitoring may serve as a warning system during periods of high solar and cosmic activity. Utilizing existing organizations such as the National Oceanic and Atmospheric Administration (NOAA) to convert solar and geomagnetic forecasts into risk indices could allow health institutions to monitor high-risk patients. Including tools such as ground-based magnetometers, spectral analysis, and heart rate variability monitoring may strengthen future research and improve comparability across studies. Recognizing space weather as a potential determinant of human health could mark a new step in preventive medicine, where monitoring cosmic conditions becomes part of protecting vulnerable populations.
